# Physicians’ knowledge and practices regarding screening adult patients for adverse childhood experiences: a survey

**DOI:** 10.1186/s12913-020-05124-6

**Published:** 2020-04-15

**Authors:** Robert G. Maunder, Jonathan J. Hunter, David W. Tannenbaum, Thao Lan Le, Christine Lay

**Affiliations:** 1 Department of Psychiatry, Sinai Health System and University of Toronto, Room 915, Mount Sinai Hospital, 600 University Ave, Toronto, ON M5G 1X5 Canada; 2 Department of Family Medicine, Sinai Health System and University of Toronto, Toronto, Canada; 3 Department of Medicine, Women’s College Hospital and University of Toronto, Toronto, Canada

**Keywords:** Childhood adversity, Child abuse, Medical history taking, Prevention

## Abstract

**Background:**

Adverse Childhood Experiences (ACEs) are common and associated with many illnesses. Most physicians do not routinely screen for ACEs. We aimed to determine if screening is related to knowledge or medical specialty, and to assess perceived barriers.

**Methods:**

Physicians in Ontario, Canada completed an online survey in 2018–2019. Data were analyzed in 2019.

**Results:**

Participants were 89 family physicians, 46 psychiatrists and 48 other specialists. Participants screened for ACEs “never or not usually” (*N* = 58, 31.7%), “when indicated” (*N* = 67, 36.6%), “routinely” (*N* = 50, 27.3%) or “other” (*N* = 5, 2.7%). Screening was strongly associated with specialty (Chi^2^ = 181.0, *p* < .001). The modal responses were: family physicians - “when indicated” (66.3%), psychiatrists - “routinely” (91.3%), and other specialists - “never or not usually” (77.1%). Screening was not related to knowledge of prevalence of ACEs, or of the link between ACEs and mental health, but was significantly associated with knowing that ACEs are associated with physical health. Knowing that ACEs are linked to stroke, ischemic heart disease, COPD, and diabetes predicted greater screening (Chi^2^ 15.0–17.7, each *p* ≤ .001). The most prevalent perceived barriers to screening were lack of mental health resources (59.0%), lack of time (59.0%), concern about causing distress (49.7%) and lack of confidence (43.7%).

**Conclusions:**

Enhancing knowledge about ACEs’ negative influence on physical illness may increase screening. Efforts to promote screening should address concerns that screening is time-consuming and will increase referrals to mental health resources. Education should focus on increasing confidence with screening and with managing patient distress.

## Background

Knowledge of the prevalence of childhood adversity and its lifelong effects on health has been advanced by the concept of Adverse Childhood Experiences (ACEs), which are typically defined to include ten types of exposure: physical, sexual, or emotional abuse, emotional or material neglect, witnessing domestic violence, permanent parental separation, or growing up with a household member with mental illness, addiction or incarceration [1]. Most adults (50–60%) report at least 1 ACE [1–3]. The more ACEs a child is exposed to, the higher the risk of many health problems, including cardiovascular disease [3–5], chronic obstructive pulmonary disease [6, 7], liver disease [8, 9], cancer [8, 10–14], infertility [15], mental illnesses and addiction [3, 16, 17]. ACEs also contribute to burden in healthcare through their association to unexplained symptoms [18], “difficult” provider-patient interactions [19], and high health care utilization [20–22]. As a result, identifying that an adult patient has experienced childhood adversity may contribute to secondary prevention of many adverse health outcomes.

In spite of their relevance to health, ACEs are not usually discussed in adult health care interactions. Both patients and doctors report that < 10% of patients are asked about ACEs [23, 24]. Patients indicate that they understand the relevance of ACEs and most do not object to being asked [23, 25]. Indeed, among patients who have experienced sexual abuse, most would like to be asked about their experience if it is done sensitively and supportively [26, 27]. Thus, barriers to discussing ACEs are mostly on the healthcare provider’s side of the interaction.

As a step towards designing interventions to encourage physicians to ask about childhood adversity (hereafter “screening ACEs”), we aimed to determine if physicians’ typical screening practice is related to knowledge about ACEs and to medical specialty. We also wished to identify the most common barriers to screening.

## Methods

We conducted an anonymous, online survey that was open to practicing physicians in Ontario, Canada. In order to reach representative Ontario physicians, we asked organizations who represent physicians of all specialties, in all geographic regions of Ontario to distribute information about the survey. These included Ontario’s five medical schools, located in Toronto, London, Hamilton, Kingston, Ottawa and Northern Ontario, and the Ontario College of Family Physicians. The University of Toronto Faculty of Medicine did not distribute the information to all faculty, but it was distributed by four of its largest departments (Family and Community Medicine, Medicine, Obstetrics and Gynecology, and Psychiatry). These organizations notified members actively (e.g. an email message from a department head), passively (e.g. a notice in a newsletter) or not at all. Two prizes of $200 were offered to random participants.

Survey questions were developed by one author (RGM) and iteratively edited by other members of the research team. We surveyed participants’ age, gender, specialty, and years in practice. To determine usual practice regarding screening ACEs we asked “How often do you ask adult patients if they experienced abuse, neglect or other serious adversity when they were children?” with five possible responses (“Routinely. I have asked almost all of my patients this at least once,” “When it is indicated. I ask patients about childhood adversity when I think it is an issue,” “Not usually. It would be exceptional for me to discuss this with a patient,” “Never,” “Other”).

Knowledge was tested by estimating the prevalence of childhood physical and sexual abuse in boys and in girls and the prevalence of all ACES (after providing a definition) on a sliding scale from 0 to 100%, and selecting which health problems from a list are associated with ACEs (ischemic heart disease, stroke, diabetes, chronic obstructive lung disease, hepatitis C, major depression, substance use disorder, generalized anxiety disorder, smoking, obesity; all have evidence of association). Participants were asked to endorse seven perceived barriers to screening from a list derived by a review of literature, by barriers voiced by colleagues at educational presentations, and by the clinical experience of the research team. The items were: not believing the information would alter care; concern that it will cause distress to patients; not feeling confident about how to ask; not having mental health resources to refer patients to if they reveal problems; concern that patients will find the question irrelevant; not having enough time; feeling embarrassed or uncomfortable; other.

Descriptive statistics were used to describe the characteristics of participants. Proportions were calculated as percentage and 95% confidence interval (CI). Relationships between variables were tested with Chi^2^ tests and analysis of variance (ANOVA) as appropriate.

## Results

Of 184 participants, there were 89 family physicians, 46 psychiatrists and 48 other specialists; 103 (56.3%) were female. The mean time practicing was 18.9 (standard deviation 13.3) years. The response rate could not be calculated because the number of physicians informed of the study is unknown.

**Fig. 1:**
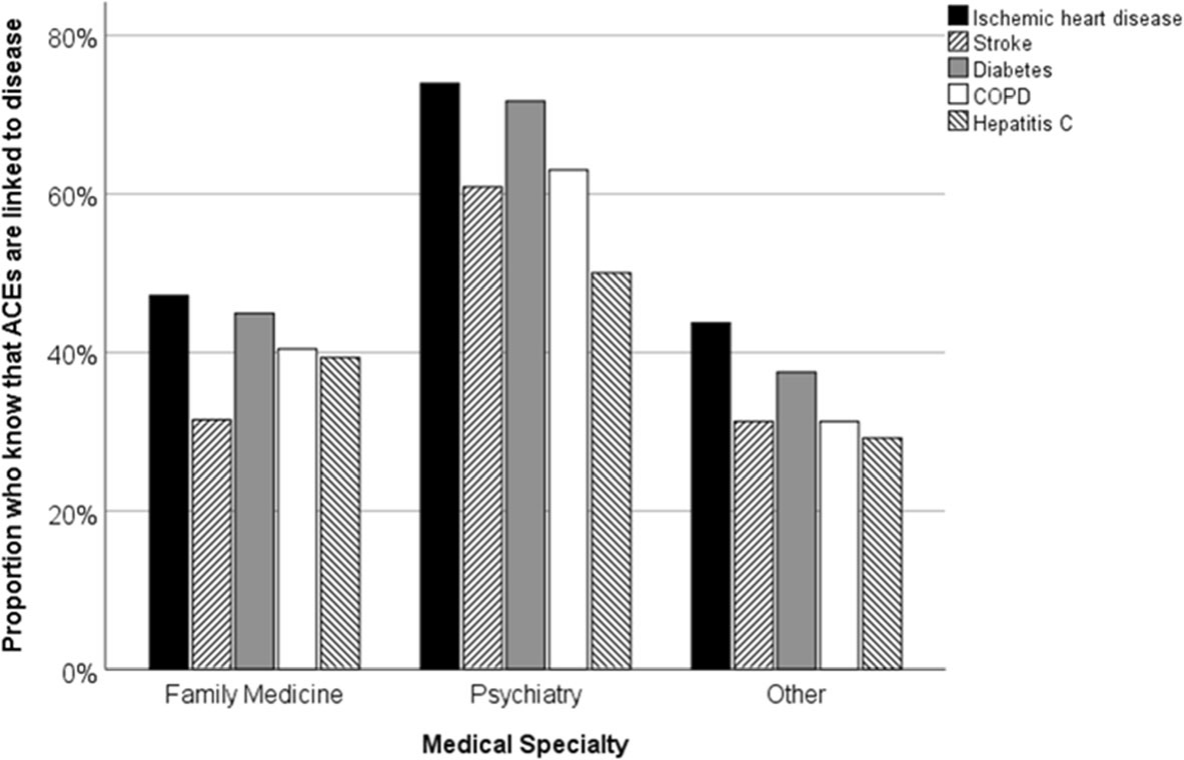
Relationship between medical specialty and knowledge that selected physical diseases are associated with ACEs. Ischemic Heart Disease: Chi^2^ = 10.9, *p* = .004; Stroke: Chi^2^ = 12.6, *p* = .002; Diabetes: Chi^2^ = 12.6, *p* = .002; Chronic Obstructive Pulmonary Disease: Chi^2^ = 10.4, *p* = .006; Hepatitis C: Chi^2^ = 4.3, *p* = .12

Estimates of the prevalence of physical and sexual abuse and of ACEs in general are shown in Table 1. Most participants knew that ACEs are associated with Major Depression (85.8, 95%CI 79.9–90.5), Generalized Anxiety Disorder (86.9, 95%CI 81.1–91.4) and Substance Use Disorder (85.2, 95%CI 79.3–90.0). Fewer knew that ACEs are associated with smoking (74.9, 95%CI 67.9–81.0) and obesity (72.1, 95%CI 65.9–78.5). This knowledge did not differ by specialty. Knowledge of the association of ACEs to physical illnesses was less common and for each physical illness tested except Hepatitis C differed by specialty, with psychiatrists recognizing the link more commonly (Fig. 1).

**Table 1:** Physicians’ estimates of the prevalence of childhood abuse and of ACEs

	Family Physicians *N* = 89		Psychiatrists *N* = 46		Other Specialists *N* = 48		ANOVA	
	*M* ± SD	95%CI	*M* ± SD	95%CI	*M* ± SD	95%CI	F	*p*
Sexual Abuse								
Girls	26.4 ± 13.5	23.6–29.2	32.0 ± 17.7	26.7–37.3	23.9 ± 12.6	20.2–27.6	3.7	.03
Boys	17.9 ± 13.4	15.1–20.7	21.2 ± 14.5	16.9–25.5	14.5 ± 8.0	12.2–16.8	3.2	.04
Physical Abuse								
Girls	29.9 ± 13.6	27.0–32.8	32.6 ± 16.3	27.8–37.4	30.4 ± 12.6	26.8–34.0	0.5	.61
Boys	30.9 ± 13.9	28.0–33.8	36.8 ± 16.8	31.8–41.8	29.0 ± 16.8	24.1–33.9	3.2	.04
Any ACEs	45.7 ± 16.5	42.2*–*49.2	49.7 ± 16.0	45.0–54.4	47.2 ± 17.8	42.0–52.4	0.8	.44

*M* mean, *SD* standard deviation, *95%CI* 95% confidence interval, *ANOVA* Difference between groups assessed by Analysis of Variance

With regard to usual practice, 58 physicians (31.7%) screen for ACEs “never” or “not usually,” 67 (36.6%) screen “when indicated,” 50 (27.3%) screen “routinely,” and 5 (2.7%) selected “other.” Screening practice was strongly associated with specialty (Chi^2^ = 181, *p* < .001); the modal response for family physicians was screening “when indicated” (66.3%), for psychiatrists “routinely” (91.3%), and for other specialists “never or not usually” (77.1%).

**Fig. 2:**
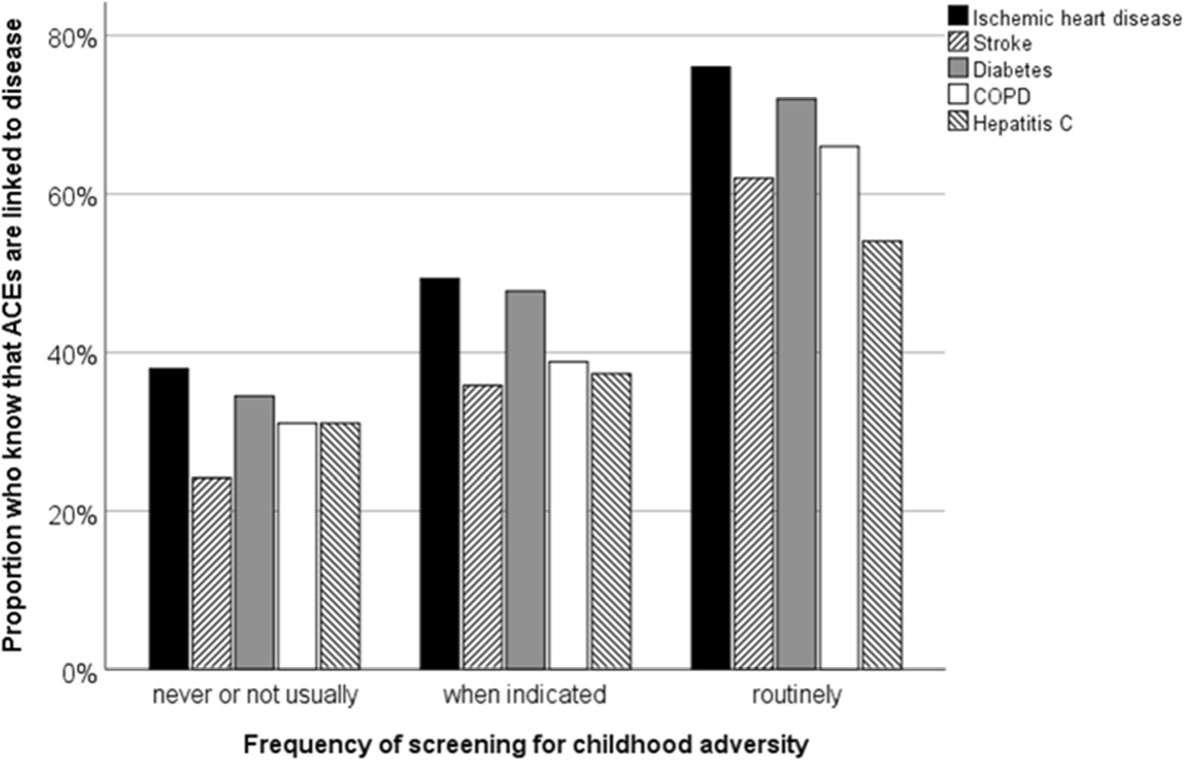
Relationship between usual screening behavior and knowledge that selected physical diseases are associated with ACEs. Five participants who indicated that their usual practice is “other” not shown. Ischemic Heart Disease: Chi^2^ = 17.7, *p* < .001; Stroke: Chi^2^ = 16.7, *p* < .001; Diabetes: Chi^2^ = 15.6, *p* < .001; Chronic Obstructive Pulmonary Disease: Chi^2^ = 15.0, *p* < .001; Hepatitis C: Chi^2^ = 6.2, *p* = .10

Screening practice was not related to knowledge of the prevalence of ACEs, nor of the link between ACEs and mental health or health behavior (data not shown). However, there was a significant positive relationship between knowledge that ACEs are associated with physical diseases and frequency of screening ACEs (Fig. 2).

The most prevalent perceived barriers to screening ACEs were not having mental health resources to refer patients to if they reveal problems (59.0%), not having enough time (59.0%), concern that it will cause distress to patients (49.7%) and not feeling confident about how to ask (43.7%). Less frequently endorsed barriers were not believing the information would alter care (41.0%), concern that patients will find the question irrelevant (30.1%), and feeling embarrassed or uncomfortable (28.4%).

## Discussion

Physicians’ screening of ACEs was related to both specialty and knowledge of ACEs’ physical health impact. Screening ACEs was routine for most psychiatrists, less frequent among family physicians, and typically not done by other specialists.

Knowledge that ACEs are associated with mental illness and addiction was widespread and unrelated to usual ACE screening practice. Knowledge that ACEs are associated with physical illness was less common and was significantly associated with screening practice. Thus, knowing ACEs increase the risk of physical illness is a potential target of education to encourage ACE screening. However, this knowledge was conflated with medical specialty. It is not known if it is knowledge or the nature of different specialty training and practices that influences screening.

Estimates of the prevalence of physical and sexual abuse differed significantly by specialty, with higher estimates reported by psychiatrists. With respect to accuracy, compared to the 2012 Canadian Community Health Survey, most physician estimates of abuse were over-estimates [3]. Physician estimates of ACEs of all types were close to what has been reported in population-based samples [2]. Thus, low rates of routine screening do not appear to be due to underestimating the prevalence of childhood adversity.

The most frequently endorsed perceived barriers were consistent with those that have been reported previously [28, 29]. This suggests another potential target for education, since available evidence suggests that screening ACEs requires less than 5 min and does not necessarily lead to new needs for mental health resources [30].

This study is limited by the method of survey distribution which precludes calculating a response rate, by self-selection of participants, which may introduce biases, and by the relatively small sample size. The study design does not allow confidence that the respondents were characteristic of all practicing physicians.

The intent of the study is built on the assumption that screening will facilitate secondary prevention and improve patient care. Discussion of ACEs in a safe and accepting circumstance may improve physician-patient alliance, as is supported by qualitative evidence from patients who have experienced adversity [26]. Asking about ACEs also supports trauma-informed care [27], for example, providing information that may reduce inadvertent re-traumatization during medical procedures. However, the assumption that screening adult patients for ACEs improves patient outcomes has not yet been directly tested.

## Conclusions

Low rates of routine screening do not appear to be due to underestimating the prevalence of childhood adversity. However, knowledge that ACEs are associated with physical illness was significantly associated with screening practice and is a potential target of education to encourage ACE screening. Common barriers to screening include the perception that it will be time consuming and will result in the need for referral for mental health interventions.

## Data Availability

The datasets used and analysed during the current study are available from the corresponding author on reasonable request.

## Abbreviations

ACEs: Adverse childhood experiences
ANOVA: Analysis of variance
CI: Confidence interval
COPD: Chronic obstructive pulmonary disease
M: Mean
SD: Standard deviation
